# Pregnancy and childbirth in English prisons: institutional ignominy and the pains of imprisonment

**DOI:** 10.1111/1467-9566.13052

**Published:** 2020-01-10

**Authors:** Laura Abbott, Tricia Scott, Hilary Thomas, Kathy Weston

**Affiliations:** ^1^ Department of Allied Health and Midwifery School of Health University of Hertfordshire Hertfordshire UK; ^2^ The Centre for Research in Public Health and Community Care University of Hertfordshire Hertfordshire UK

**Keywords:** pregnancy, institutions, childbirth, prisons, Goffman

## Abstract

With a prison population of approximately 9000 women in England, it is estimated that approximately 600 pregnancies and 100 births occur annually. Despite an extensive literature on the sociology of reproduction, pregnancy and childbirth among women prisoners is under‐researched. This article reports an ethnographic study in three English prisons undertaken in 2015‐2016, including interviews with 22 prisoners, six women released from prison and 10 staff members. Pregnant prisoners experience numerous additional difficulties in prison including the ambiguous status of a pregnant prisoner, physical aspects of pregnancy and the degradation of the handcuffed or chained prisoner during visits to the more public setting of hospital. This article draws on Erving Goffman's concepts of closed institutions, dramaturgy and mortification of self, Crewe *et al*.'s work on the gendered pains of imprisonment and Crawley's notion of ‘institutional thoughtlessness’, and proposes a new concept of institutional ignominy to understand the embodied situation of the pregnant prisoner.

## Introduction

A central theme in the extensive sociology of pregnancy and childbirth concerns the experiences of child‐bearing women (Oakley 2016); however, this literature has overlooked the experiences of women in prison. British midwifery values in the 21st century encompass bodily autonomy and choice (Sandall *et al*. 2013). Women imprisoned during pregnancy and/or childbirth clearly experience more intense challenges to their choices than their non‐incarcerated sisters. The circumstances of pregnant prisoners contrast starkly with best midwifery practice where current practice regarding empowerment, continuity of care, partnership models, support of physiological birth and choice of birth location should be guiding principles (McCourt *et al*. 2006, Sandall *et al*. 2013). This article addresses perinatal experiences of prison through the descriptions and narratives of women, staff and researcher field notes.

The prison population of women in England is approximately 9000 (Ministry of Justice, 2019). Surprisingly, pregnancy and births numbers are not recorded but estimated at approximately 600 pregnancies and 100 births per year (Abbott 2018, Kennedy *et al*. 2016). A review of the UK female prison estate in 2007 followed reports of several suicides in 2006–2007 (Corston 2007). The analysis demonstrated that most women in prison were disadvantaged either through poverty, mental illness, historic abuse or addiction, that the majority had children, and that several were pregnant. Of the 12 women's prisons in the UK, six have Mother and Baby Units (MBU), with 64 MBU places available nationally (Ministry of Justice 2019). Pregnancy is often discovered during initial health assessments on reception to prison (Corston 2007, Gullberg 2013). United Kingdom statute requires that all prisoners should receive equivalent health care to that provided in the community (Council of Europe 2006; Rogan 2017). However, there is no known requirement for midwives to be positioned as permanent staff members in prison healthcare departments and no specific UK prison service mandatory guidance for staff when dealing with pregnant women. Guidance on the management of women prisoners indicates that pregnant women should receive suitable nutrition and rest, handcuffs should not to be used after arrival at hospital and they should not travel in cellular vans (National Offender Management Services 2014). The guidance directs the prison service to make adequate provisions for women wishing to breastfeed their babies and suggests that careful planning should take place when women are being separated from their babies due to the risk to their mental health.

## Review of the conceptual literature

Goffman's concept of a total institution is beneficial to an understanding of the prison system and power relationships (Goffman 1961). Definitions of prisoners as described by staff, placed value judgements on inmates, separating ‘them’ (prisoners) from ‘us’ (‘normals’) and help to explore the concepts of dehumanisation, othering and staff/prisoner relationships. Goffman's (1959) description of the ‘mortification of self’, although not specifically related to women, illustrated how a prisoner is given wearable ‘marks of shame’ (e.g. handcuffs). In prison, women's psychological pain is often demonstrated through self‐harm behaviours (Chamberlen 2015, Walker *et al*. 2016). Institutional spaces were described by Goffman (1961) as segregation and emotion zones eliciting a wide array of emotions. Feeling dehumanised and the sense of losing personal identity have also been defined as synonymous with the experience of being a prisoner (Halliday *et al*. 2017, Zimbardo 2016).

Sykes’ work (1958/2007: 68) developed the concept of ‘pains of imprisonment’ exposing the deprivations experienced by men in prison, including: the loss of goods and services; relationships; autonomy; security and liberty. Sykes (1958/2007: 78) suggested that the loss of liberty is not restricted to physical deprivations, but rather represents a ‘loss of status’ and described how material deprivations lead to difficulties in sustaining health. Although equivalence of care is a current policy requirement (Rogan 2017), it is argued that health care blends into prison culture, with healthcare staff adopting prison values (Ross *et al*. 2011). The loss of autonomy as seen through Sykes’ (1958/2007: 73) lens suggests a ‘total and imposed’ helplessness.

Crewe *et al*. (2017), building on Sykes work, identified the ‘gendered pains of imprisonment’ with women being more likely to be affected by loss of privacy, autonomy and control than male prisoners. Their research on life imprisonment exposed greater implicit understanding of the gender differences among lifers. It appeared that women often experienced greater suffering than men due to the experience of childhood abuse, which exacerbated the painful loss of autonomy, relationships and security. Crewe *et al*. (2017) found that loss of control was a greater pain for imprisoned women than men, triggering a sense of crisis, exacerbating stress levels and subsequently affecting the ability to maintain mental wellbeing.

## Empirical studies

There is limited qualitative research exploration of pregnant women's experiences of incarceration. Much of the literature has relied on scoping exercises and views of prison staff, rather than those with lived experience (Albertson *et al*. 2012, Edge 2006, O'Keefe and Dixon 2015, Price 2005). Fritz and Whiteacre (2016) found that the healthcare needs of incarcerated pregnant women were often left unmet, with negative experiences of antenatal care, intensified by the loss of control over their pregnancies. The literature reveals women's accounts of connectedness with their unborn baby (Chambers 2009, Wismont 2000) and anticipatory grief when pre‐empting separation, compounded when separation actually occurs (Gardiner *et al*. 2016, Schroeder and Bell 2005). Limited access to support, pre‐natal education and suitable nutrition was demonstrated by Ferszt and Clarke (2012). The Rose Project, undertaken in Scotland, pursued views of pregnancy and becoming a mother in prison, and the constant theme of separation from the baby (Gardiner *et al*. 2016). Recent research in the UK described the complications involved in gaining a place on an MBU and the complexities of the appeal process when denied a place (Abbott 2018, Sikand 2017). Previous qualitative health research has often focused on narratives without observing the milieu (Liebling 1999).

This article builds on the conceptual work of Goffman (1959, 1961), Sykes (1958/2207) and Crewe *et al*. (2017). It proposes a new concept of *institutional ignominy* to capture the depth and complexity of the experience of pregnant prisoners.

## Methods

With the setting so intrinsic to the imprisoned pregnant woman's experience, ethnography was selected in the current study as the methodological approach to understand fully those experiences, viewed through the subjective lens of midwife/prison researcher (Hammersley and Atkinson 2007). This study set out to understand the experiences of pregnancy by interviewing women and prison staff and by non‐participant observation. By taking an ethnographic stance, the research offers a unique perspective of the prison experience, and one which has not previously been undertaken by a midwife researcher.

The study aimed to explore women's experiences of pregnancy in prison through qualitative interviews with a sample of women prisoners, a further sample of prison staff and observational field notes. Favourable ethical opinion was granted by the National Offender Management Services (NOMS) on 25 September 2015 (approval number: 2015‐209) through the Health Research Authority Integrated Research Application System (IRAS). A 2‐year period of training and negotiation led to access being granted to three prisons: a closed prison without an MBU attached; a closed prison with an MBU attached; and an open prison with an MBU attached. An application to bring in an encrypted digital recording device (a prohibited item) was completed and permission was granted by managers given to audio‐record interviews. For a full description of the methodology please see Abbott (2018).

## Sample, recruitment and fieldwork

Each prison compiled a list of pregnant women but, as pregnant women were not housed on one prison wing, seeking out potential participants was often a complex task. Staff helped to locate the pregnant women and a movement slip was issued to women who agreed to have a preliminary conversation. This permitted each woman time away from activities to receive, read and discuss the approved participant information sheets. In total, 28 women consented to participate in audio‐recorded interviews: 22 while incarceration and six following release from prison. Two women declined to take part in the study. Five of the women who remained incarcerated agreed to follow‐up interviews. Ten staff members consented to audio‐recorded interviews, including six prison service staff and four healthcare personnel. Data were collected over a 10‐month period including 58 audio‐recorded interviews and written field observations. All fieldwork was undertaken by Abbott (2018) who is a registered midwife.

The skills of navigating the prison system and communicating with women and staff were developed prior to commencing fieldwork and during the pilot phase. Spending over 260 hours in the prison setting meant that women and staff alike got to know the lead researcher. Field notes were made in a total of 25 notebooks, recording the minutiae of prison life and descriptions of the prison milieu, for example ‘the smell of sour milk; the tension, the thick air, the angry atmosphere, it's claustrophobic. I want to get out of here; it feels oppressive’. Capturing the atmosphere through description and reflection gave context to the women's experiences.

Most interviews were an hour or more in length, undertaken face to face and in private. It was commonplace for a woman to be summoned back to her room, so hasty goodbyes were the norm. Maintaining confidentiality and anonymity were paramount, not only to protect participants but also due to the potentially sensitive exposure of women whose case or trial could have media coverage. Interview transcripts were anonymised by removing real names or geographical locations. The transcripts were initially reviewed, and primary notes were made about characteristics and any individual differences from field notes gathered at the time of the interview. As audio recordings were deleted immediately following transcription field notes of the nuances, body language, appearances and interruptions provided valuable reminders of the milieu. In prison research, reflexivity and taking an auto‐ethnographic stance are said to be critical in order to maintain a stable‐state of mind and to increase objectivity (Crewe 2009, Jewkes 2012, Liebling and Maruna 2013). This was especially true in situations where women's stories were distressing in an environment which is punitive by nature. Indeed, Lofland and Lofland (2006) warned against the potential for outrage that a given situation may generate. Writing a reflexive diary through fieldwork was indispensable and following each visit, thoughts and reflections – totalling approximately 60 separate entries – helped to unravel a sense of feeling misplaced in an alien environment.

## Analysis

Thematic analysis was undertaken to explore the interview data and observational field notes (Hammersley and Atkinson 2007, Ritchie *et al*. 2013). A line‐by‐line approach to transcript review allowed submersion in order to see patterns in the narratives (Barbour 2013). NVivo, a computer‐assisted qualitative data analysis software package (CAQDAS), supported early manual coding providing an iterative data review process and theme reduction towards the end of fieldwork. The iterative process of reviewing the data and reducing the themes commenced towards the end of the fieldwork. A total of 736 nodes were initially identified linked to 72 categories. Ensuing modification condensed the data to a more practical 178 nodes relating to 24 categories. To avoid duplication, some nodes such as ‘room descriptions’ were transferred to a subcategory of ‘environment’. Emotion was then relocated under the central theme of ‘coping’; ‘handcuffs’ became one of the 13 child nodes under the parent node ‘stigma’ which was relocated under the central emergent theme of ‘institutional ignominy’.

This article focuses on the interviews with the women and draws on interviews with staff and material from the field notes to provide context for the women's experiences. Pseudonyms are used throughout.

## Findings

The following analysis draws on the interviews with women and staff combined with observations of the prison setting to consider whether pregnancy grants women prisoners a special status, aspects of the physical pregnancy and the degradation of the pregnant prisoner.

## Special status?

It was apparent that the women prisoners interviewed distinguished themselves as different from the normal prison population. For many participants, pregnancy appeared secondary to their prisoner identity through feeling a loss of control and subsequent disempowerment. Women would commonly try to consciously ‘block out’ their pregnancy. The reasons for this were complex, but pregnancy denial appeared a way of coping with the ‘horrible’ experience of being pregnant in prison:

‘‘You know, although you are pregnant, a part of you forgets that you are pregnant. Because you're in there because there's a lot to deal with. It's terrible really because you don't focus on what you should be focusing on. It all goes out the window’’ (Jane).

Several women experienced an increasing sense of fear as their pregnancies progressed to an inescapable visibility. Trixie recalled how another inmate had threatened her with violence:

“She was shouting at me, and I tried to shut the door and she slammed the door, like opened it on me, so I had to quickly catch it and hold the door shut. And she kept trying to push the door on me, and I just told her to go away and she just kept saying that … ‘I wish your baby dies’” (Trixie).

The threats Trixie experienced left her feeling afraid for the safety of her baby particularly in her own room with limited means of escape.

Some women expressed anger at not having special status as a pregnant prisoner. Some pregnant women perceived that their pregnancy should provide protection against prison life: ‘I don't think they should put pregnant people with four other people in the room’, yet the distinct status usually afforded to pregnant women in mainstream society was mainly absent in prison. For pregnant women to be treated the same way as non‐pregnant prisoners appeared to participants an additional pregnant pain of imprisonment – their pregnancy afforded no special treatment. A common experience among all participants was their sense of injustice when they did not receive perceived entitlements as pregnant women, such as healthy snacks or suitable bedding.

The relationships between prison officers and pregnant women have been previously described as distorted due to potential conflict between caring and custodial duties (Abbott 2019). Having a mutually positive relationship often relied on women's compliance. Nonetheless, language used by staff often appeared to conflict with support and encouragement, for example in the homogenous label of ‘the pregnants’. This role confusion resonates with the criminology literature where attributes of prison officers were habitually inherent to the prison experience (Crewe *et al*. 2015). Labels are intended to differentiate the needs of prisoners but also serve to further strip their personal identities. The colliding ideologies of custodial prison officer and the occasional default to carer of pregnant women is both curious and alarming especially where the prison officer is required to shift their relationship with the pregnant prisoner at various points throughout her gestation. Such paradoxical interaction, custodial/caring, challenges what the term ‘relationship’ means in the institutional setting and renders the pregnant woman vulnerable due to lack of consistency should the prison officer not subscribe to the pregnancy needs of the woman.

The demonstration of empathic responses towards pregnant prisoners and dedication to the prison officer role was commonplace (Abbott 2019). Staff interviewed were often supportive and compassionate: ‘Some women want officer support, If they've got no family and all they've got is a uniformed member of staff stood at the side of them, I think it must be so distressing to go through that on her own, without that family support and to be with us’; yet, there was a recognition that the staff / prisoner relationship had limitations and firm boundaries were necessary:

“They are not real relationships … we leave them [women] at the gatehouse at the end of a shift … But it must be hard for them, they can't plan, because it's all governed by being told what to do in prison … it must be worse in here, because you can't just go out and get in the car, get on a bus, pick a phone up” (Sandra, Prison Officer).

The perception of relationships between officers and pregnant women of being distant, despite having such an intimate presence in their lives, contrasts with the midwifery connection with a woman whereby any engagement, however transitory, is always seen as a relationship (Bradfield *et al*. 2018).

## The physical pregnancy in prison

The concern for special status may have been directly related to women's perception of their unborn babies’ suffering as all participants felt deprived of appropriate food. One respondent claimed that: ‘You don't get enough fresh fruit, fresh veg that you need’. Normal, physiological symptoms of pregnancy were exacerbated for most participants: ‘I had to swallow my sick just to get an officer to open my door’. Abi's, symptoms of nausea and vomiting were so extreme that she had been hospitalised twice with hyperemesis gravidarum (HG). During each interview, she sank down into a chair, arms held tightly around her body or with her head in her hands, looking pale and sad:

“I really feel ill; it's horrible, and they just still make you do things. They must think, oh … you're pregnant, you're going to feel sick. No, I promise I feel really ill, and I can't eat. I wish I could eat! I'm scared that my baby will die or something, because it won't get no food” (Abi).

Several women experienced nausea and vomiting which, although unpleasant, are normal physiological responses to pregnancy and usually not debilitating. However, Abi, suffering from HG, found that her extreme bodily symptoms were often disbelieved by staff, despite her losing weight, her relentless vomiting and inability to eat anything or even to swallow her own saliva. Kayleigh had been imprisoned several times previously but not as a pregnant prisoner. She expressed anger at her treatment as a pregnant woman, demonstrating a shift in her identity due to her protectiveness towards her unborn baby saying:

“Just the fact that I'm pregnant, and the way that they are operating, it stinks! If I wasn't pregnant, I wouldn't care. If I wasn't pregnant, I would do my time, right? But I am pregnant, there is another little human being in here who I've got to take care of. That's how I've got to think of it now, I'm not on my own. I am number two, this is number one” (Kayleigh).

Women used baggy clothes to hide their pregnancy and so blend in and not draw attention to themselves, or to ensure they felt protected from harm from other prisoners:

“I've got baggy tops, so I just always have to hide my bump, and like most people couldn't recognise that I'm pregnant, so that's a good thing. So, I'm glad I'm not like out here [gestures] I want it hidden, because I don't know who's who and who is in for what.” (Lola).

Some staff expressed their views about the environment from a pregnancy perspective, demonstrating empathy towards the women. Yet, interestingly, the label of ‘pregnants’ was uttered unprompted in two of the prison settings:

“Things like clothing are a big issue for pregnants. The IEP system [incentives and earned privileges] policy only allows so many kit changes and catalogue providers do not provide maternity wear or bras and women cannot afford to buy the expensive bras needed” (Elaine, Prison Officer).

“The pregnants are supposed to get extra food and sleep on the bottom bunk but it doesn't always happen” (Jenny, Prison Officer).

However, despite the dehumanising label, sympathy for women was also universal to each site:

“Prison is not the place for a pregnant woman” (Janet, Prison Officer).

Entries from field diaries exposed the environment and gave examples of smells, sounds and general observations:

“There's a long corridor with rooms either side; it smells very strongly of tobacco, it's very smoky. One of the women I've been interviewing has a room there. There's no fresh air” (Fieldnotes, November 2015).

Alongside the sensory overload from noise, the need for fresh air is a key feature of the prison experience, and this was also depicted in field notes as a description of taking a ‘deep gulp of air’ on leaving the setting. The sense of air hunger, due to the lack of fresh air or not having windows to open was especially difficult for pregnant women. The sense of having ‘no control’ over one's life or pregnancy generated distress among all participants: where they would give birth; who might support them; anxieties regarding receiving medications previously prescribed in the community; and ultimately whether they would be allowed to remain with their babies beyond delivery. Prison life demands that a woman is dictated to concerning when and what she eats, when she sleeps, what she drinks and when she accesses health care. The sense of loss of autonomy is, of course, held common with all prisoners (Crewe *et al*. 2017); however, the pregnant woman experiences unique and multiple fears. For most participants, the lack of privacy reinforced a feeling of degradation:

“There is no privacy … officers can come into my room at any time. If they decide they want to unlock the door to come in for a reason to … I'm used to it; I don't like it, but I'm used to it” (Caroline).

Caroline's acceptance, of being ‘used to’ such invasion of privacy, juxtaposed with the fear of labouring in the presence of strangers, typified the uncertainty and ambiguity which many of the pregnant women felt about their prison experience. Women would often express the most ignominy and annoyance when asked in interview about their treatment as pregnant women. Feeling ‘sub‐human’ led some women to worry about labour and birth, amid fears of not being heard or cared for when relying on officers:

“I don't really want them to care, but as a human being you should at least have a little bit of concern about a pregnant woman” (Trixie).

Trixie expressed her feelings of ambivalence of not wanting to be cared for, yet feeling that it was the duty of staff to care. Conversely, prison officers often expressed their feelings of compassion towards the women:

“The courts have punished them by being here, and it's our job and our duty even to look after them. Even more so when they're pregnant … you kind of want to wrap them up a bit in cotton wool” (Ruby, Prison Officer).

The sense of disempowerment at the prospect of being left unsupported in labour and fear of not being unlocked in time for transportation to the hospital for the birth were distressing for all women who were spending their entire pregnancy in prison. This sometimes influenced a woman's choice regarding the mode of delivery of her baby. Strikingly, some women chose a medicalised birth, such as a planned caesarean section, over the uncertainty of the potential complexities around spontaneous labour in prison to gain agency. The environment was perceived as so hostile to labouring spontaneously that, for Trixie, it felt unsafe to go into labour in prison. Indeed, the findings demonstrated that some women attempted to regain agency by requesting a medical birth:

“I told the doctor: ‘I'm not having a natural birth’. I said, ‘I'm not going to have it’ … especially the way they treat you here, I'm not going to put myself in danger. I'm certainly not going to put my baby in danger. I'd rather be in the hospital in safe hands” (Trixie).

Birthing choices gave some women a sense of control over the timing of birth and lessened the fear they had of commencing labour in prison.

## The degradation of the pregnant prisoner

Women talked of ‘not showing any weakness’ and therefore ‘covering emotions’ with what Hochschild (2012: 56) termed ‘deep acting’ when outside of their rooms yet reverting to feelings of isolation when in their cell where they would: ‘sit there and cry’. The ambivalence of the woman's status of prisoner and pregnant prisoner meant having to moderate emotions especially frustration and anger which was difficult for some women:

“To get anything then you need to stamp your feet like a toddler. You need to create and make a big scene and make a big fuss. Well, I was in a predicament as if I'd have started doing that, then I would have been seen as aggressive. Which then, in turn, I wouldn't have got my place on the Mother and Baby Unit, and I wouldn't have been able to keep my daughter” (Layla).

Women recognised that the denial or suppression of their emotions was potentially causing them mental harm. On the one hand, Layla had feared that if she made a ‘scene’ she could lose her daughter yet having to be ‘respectful and polite’ translated to passivity. Layla blamed her own submissiveness for the fact that subsequently her labour was ignored and believed that her compliance was perceived by staff to translate as someone who ‘wasn't bothered’. Leakage of breast milk led to clothing stains with a common recommendation from staff to: ‘rip a sanitary towel in half’ to soak up excess milk. While normalised by staff, women described their shock at not being able to purchase breast pads or to have a supply of them in prison.

Women felt the frustration of being dehumanised and being characterised exclusively as a prisoner: ‘I want them to know me as a human being’. Most women described how they ‘don't get treated like a person’. Institutional props which contributed to a sense of dehumanisation in the form of keys, handcuffs and chains served to further strip away their identity and self‐worth. There was a perception that handcuffs gave the officers, whether female or male, power over the woman: ‘it's like they are my keepers’. The gaze of women, their partners and children in maternity departments left pregnant women feeling judged and shamed in public:

““I just felt like I was being watched and because they are in uniform and I am in handcuffs it's like they have some kind of control over you, so you can't express yourself in a normal way like a normal pregnant woman would. It's just so degrading” (Jane).

Women who had attended hospital appointments accompanied by officers and in handcuffs would talk about how the public would look at them: ‘it's other people looking at you, judging’. Lola's experience of being handcuffed intensified her humiliation and sense of feeling judged by society:

“I'm handcuffed to an officer in prison uniform, and I'm pregnant and everybody is looking … and you can see people, they think ‘what has she done?’ People shouldn't judge people; they should listen to your story first” (Lola).

All the women interviewed talked about the shame of being publicly observed as a pregnant prisoner. Women described feeling like: ‘a number’ or ‘marked with the same card’. Research field notes demonstrated the dehumanising language of prison:

“The shout of the prison officer, it being ‘bang up time’. The noise, clatter, clinks and shouts of ‘behind your doors ladies’, making you jump no matter how many times you hear it said. Being called ‘Miss’ is not normal for me, I ask women to call me by my name” (Fieldnotes, December 2015).

Women often described their treatment by officers as lacking humanity; however, this contrasted with the staff view that they believed they were treating the women with care. In common with the general prison population, being visible in hospital and being handcuffed and/or accompanied by prison officers caused anxiety (Easton 2018, Wahidin 2004). Hospital appointments were an essential and regular occurrence, yet most of the women spoke of wearing handcuffs or chains in the maternity department. The experience of being repetitively moved from one closed institution to another, while visibly pregnant, generated humiliation (Abbott 2019). All pregnant women in this sample described the experience of being handcuffed as demeaning. Susan described her experience of shame:

““It's really embarrassing, being cuffed – sometimes they uncuff you when you get to maternity, because there's other pregnant people there that are all anxious. I had to sit in reception handcuffed and everyone, everyone that was coming in and out was just looking down at me” (Susan).

Interviews with staff demonstrated the compassion felt towards pregnant prisoners, yet data analysis of the language used by Prison Officers in two of the prison settings established several dehumanising labels such as: ‘the pregnants’. This juxtaposed the sanitised description of ‘our pregnant residents’ in the third ‘open’ prison, where officers considered the women more as guests than prisoners, giving a more humanised, albeit neutral, label. These labels suggest objectification and therefore an element of dehumanisation. The way women described their treatment highlighted how they valued being ‘treated like a human being’. Yet, regardless of the type of setting, data analysis demonstrated that shame and humiliation were emotions articulated across all three prison estates.

Being handcuffed and placed in chains, or being strip searched by female officers, as Cleo experienced on return from hospital: ‘they made me shake out my pad … and squat’, were especially dehumanising experiences. Tammie described her experience of being sentenced to prison 3 weeks after having birthed her son: ‘Even animals are treated better’. Animal metaphors were common expressions used in relation to the prison experience: ‘animals get six weeks with their Mum before they are taken, my baby only got three weeks with me’. Jane compared her experience of visiting the hospital to being like a ‘dog on a lead’ explaining how her identity as pregnant prisoner was controlled leaving her with a sense of disempowerment:

“I didn't feel I could ask them to leave as at the end of the day, they've just brought me in handcuffs so how could I say to them ‘oh could you just leave’. You're basically controlled and I don't know how to explain it but it's like you are a dog on a lead and you have only got so much before they are pulling you back” (Jane).

The most severe form of degradation occurs when a birth takes place in a prison cell. In the UK it is a legal violation for anyone other than a Registered Midwife or Medical Practitioner to attend women in childbirth, except in ‘sudden or urgent necessity’ (Nursing and Midwifery Order 2001). Layla went into spontaneous labour in prison, three and a half weeks early, and felt her status as a prisoner over‐rode her status as pregnant woman. She was assessed by nursing staff who were not trained in midwifery and therefore unqualified to recognise either labour or that she required assessment in hospital due to several risk factors.

Layla shed her cervical plug and was sent to health care where she saw a nurse, who estimated that she had another 7 to 10 days before birth. Layla was particularly concerned because, in her previous pregnancy, loss of her mucous plug had signalled the start of labour but her attempt to explain this to staff was met with indifference: ‘They were like … we'll sort that out when and if you go into labour’. She began to have contractions that night and thought she was in labour. A nurse came to her cell, examined her abdomen and told her that she was not in labour. Layla accepted her position of powerless prisoner, rather than labouring woman. Within 10 minutes of the nurse's leaving her waters had broken and she rang her bell again. Layla described the nurses as then being in ‘absolute panic’ saying, ‘We need to get an ambulance, we need to get her to hospital! I says, I haven't got time to get to hospital. I did say to you I was in labour’. Layla expressed her feelings of humiliation and disempowerment due to the repudiation of her labour and described how she gave birth to her daughter:

“I was laid there on my bed, in my cell with a male nurse and a female nurse, not midwifery trained and then out popped [baby] at twenty past one. Still no ambulance, still no paramedics and she came out foot first” (Layla).

The lack of recognition by health and prison staff of the limitations to their sphere of responsibility led to increased suffering for Layla, already in terror of not knowing whether she would keep the baby or not, assuming survival. The loss of privacy compounded the loss of dignity and decency, intensifying the experience of institutional ignominy.

## Discussion

The following discussion expands upon the themes of: special status; the physical pregnancy and degradation; pregnancy as an existential crisis; and the key overarching concept of institutional ignominy. The conceptual framework for this article is derived from Sykes’ (1958/2007) ‘pains of imprisonment’ and Goffman's sociological examination of closed institutions, dramaturgy and mortification of self (Goffman 1959, 1961, 1968). The present study questioned whether and how these pains were understood and experienced by pregnant prisoners. Sykes (1958/2007: 77) stated that the prisoner ‘can never feel safe … he is evaluated in public view’. The sense of embodiment for the pregnant prisoner juxtaposes pregnant women's embodiment in free society. It is normal for a pregnant woman to want to seek out privacy and retreat from public view because pregnancy can be embarrassing due to biological changes and bodily prominence (Longhurst 2001).

In mainstream society pregnancy is usually respected as a ‘special social category’ (Balin 1988, Molina 2019). For pregnant women in prison, it appeared that there was a disregard for their unique pregnancy status. The shame of being in prison compounded impending motherhood could leave her with a perception of being blamed by society and stigmatised due to the visible mark of pregnancy. The constant cyclical reprocessing and re‐exposure to stigma through public display highlighted the transgressive nature of being both pregnant and a prisoner.

## The incarcerated pregnancy as an existential crisis

For pregnant women in prison, it appeared that the special status of mother‐to‐be was disregarded. In contrast, in prison, this status was superseded by being a prisoner and, hence, a criminal (Goffman 1968): ‘I shut it out [pregnancy] because I was in prison’. Goffman (1968: 24) describes this universal pain of imprisonment as ‘mortification of self’ in which the first stage, the barrier between prison and the outside world, equals the first curtailment of self.

Leder (2016: 209) noted the public perception of a prisoner as a ‘social caricature as savage, bestial and sub‐human’. Being pregnant necessitated regular public trips to attend recurrent hospital appointments, a different experience from other prisoners. Women were usually not forewarned about the timing of external appointments therefore had no time to physically or mentally prepare. They expressed their perception by the public as monsters and murderers in the antenatal department and described how mothers pulled their children closer to them as the public stared.

An autonomous identity is said to be essential in order to be ‘related as one human being to another’ (Laing 1960: 46). The process of othering (Brons 2015, Canales 2000) came not only from staff towards prisoners but, interestingly, from pregnant prisoners towards non‐pregnant prisoners, too, with exclamations of: ‘I'm not like them!’. The expectation of different treatment, especially in relation to food and health care, was often coupled with incredulity, for example ‘I am getting the same as normal prisoners!’. The pain of dehumanisation was common in the pregnant women: being viewed as ‘just a prisoner’ and being afforded no ‘special treatment’ was distressing. The experience of pregnancy in prison builds on Sykes’ and Crewe's descriptions of pain and suffering, whereby the loss of autonomy and control leads to a loss of choice for the pregnant woman in English prisons. Not knowing whether she would keep her baby led to a painfully ambiguous state.

## Punishing the imprisoned pregnant body

Women's physical agency in prison encompasses two specific aspects of pregnancy: bodily concealment and control over her birth choices. The pregnant woman appears to endure additional constraints as she attempts to exert control over her condition. Bodily cravings for food, comfort and safety were often ignored within the prison system. For most women, as their pregnancy became more visible, were marked with what Goffman (1961:14‐15) described as ‘tribal stigma’, an undesired difference from the normal pregnancy experience. Pregnancy is usually celebrated in free society but women in prison sought to hide their growing bodies, disliking their visibility of difference and belonging to a minority group: ‘I try to hide my bump … I wear baggy clothes’. This represents a dichotomy where the dualistic elements of sacrosanct (pregnancy) collide with the profane (criminal) elements of social life. Leder (2016: 175) suggests that the prisoner's body is viewed as a ‘possession of the state’. Overwhelmingly, most women experienced bodily suffering during their pregnancy, often brought on, or exacerbated by, institutional thoughtlessness (Crawley 2005). Women's leaking breasts served as a visual reminder of women separated from their babies. One participant described her observation of witnessing another woman's uncontrolled leakage of breast milk as like ‘bleeding all over the place’. Abi was so unwell that she could not work, and refusal led to loss of possessions and privileges which temporarily distracted her, such as a television. The prison environment is often described negatively (Zamble and Porporino 2013) but the question of the impact of such a difficult setting on the pregnant woman has been largely unexplored. Sykes’ (1958/2007) depiction of the helpless prisoner being thrust into past childhood pains resonates more deeply with imprisoned women whom Crewe *et al*. (2017) describes as being in ‘psychological limbo’.

Knowing whether a woman will be unlocked in time for transfer to hospital when in labour is a pain of imprisonment unique to pregnant women. Medicalised modes of delivery were chosen by Abi and Trixie and offered a sense of control, although for Trixie, even as she approached the latter stages of pregnancy, the procedure date remained unknown. Sykes (1958/2007) argued that the purpose of prison is to remove liberty and exert control yet, in pregnancy, these pains are exacerbated threefold: loss of bodily control; loss of control over her pregnancy and birth choices; and ultimately loss of control over whether she could be a parent to her unborn baby. Layla, who birthed in her prison cell, suffered particularly degrading treatment, having her right to privacy and dignity removed in addition to being unable to make choices over her body and for the safety of her baby.

## Institutional ignominy

The expression of shame was a probable expectation, given previous research on concepts of stigma as described by scholars such as Goffman (1961). Nonetheless, these findings reveal that ignominy intensified as an institutional response to pregnancy as women felt paraded in public while branded with prisoner emblems. The inner torment this caused women was often expressed in indignation in that the public opinion that they were failing as a mother was unwarranted. The term institutional ignominy captures the distinct experience of pregnancy in prison overall, unique amongst accounts of incarceration. A key finding in this study was the impact on the women who oscillated between two institutions – prison and hospital – involving concealment of pregnancy in prison and public display in hospital. A central theme running through this research was shame causing painful humiliation for the pregnant woman.

We argue that Goffman's concept of mortification of self does not fully capture either the tensions inherent in the ambivalent status as a pregnant prisoner or the sense of recurrent movement between institutions as a pregnant woman. This article proposes a new concept of institutional ignominy to extend Goffman's concept of mortification of self and to capture the complexity of the pregnant prisoner, her ambivalent status and the distinct experience of pregnancy whilst ‘locked up’. The process of moving between the two settings, adorned with symbols of restraint (handcuffs / chains) and accompanied by uniformed authority figures (prison officers) reinforced institutional ignominy, a situation in which the double degradation came with the artefacts of power and control leading to the excruciating feeling of being judged as both pregnant and a criminal/prisoner. Layla's birth in a prison cell fused the concepts of institutional thoughtlessness (Crawley 2005) and institutional ignominy. Her experience of birth depicted what Sykes (1958: 77) describes as a ‘fight for the safety of (her) person … a tense and fearful existence’. The denial of Layla's basic needs as a woman in labour was extraordinary, symbolic of mortification of self (Goffman 1968), breaching her human rights (Van Gundy and Baumann‐Grau 2016). While Layla was ‘thoroughly in her body’ (Laing 1960: 69), exclaiming, ‘I know my body!’, staff negated her sense of embodiment: ‘you are not in labour!’, causing Layla to deny her physicality and feel abandoned and disempowered. Such vulnerability disrupted Layla's sense of safety in that her bodily autonomy was both invalidated and violated (Laing 1960: 44).

In common with other research findings (Crewe 2009, Harvey 2008), prisoners in this study found ways to mask their pains, often retreating to their cells at night‐time where emotions would flow. The wish to blend in with the main prison population in order to avoid being singled out for attention or occasional threats of violence, intensified women's stress. The effort of masking stress led women to be concerned about the effect on their unborn babies. Being ‘front of stage’ (Goffman 1959) as a pregnant woman yet having this ignored by some staff who viewed her as just a prisoner was emotionally very difficult.

The societal signals and visual codes of behaviour towards pregnant women seemed not to be applied in prison. The tension involved in restraining emotions for fear of the ultimate punishment of losing one's baby through enforced separation generates considerable strain and physical burden upon pregnant women. The women experience turbulent emotions, from denial to despair to the isolation of crying at night. During the interviews, prison staff acknowledged their difficult emotion work, especially when supporting women separating from their babies. The identity of the pregnant prisoner was often confused and symbolised dualisms as the societal status of being a pregnant woman was superseded by the prisoner/criminal label. Women reported feeling stripped of their identity, and staff often found it difficult to see the pregnancy as part of her uniqueness, preferring to keep the prisoner groups homogenous. Restraint is shown by these women who mask the physical and emotional bond with their unborn baby and appear to experience a depth of pain beyond the ordinary tensions of incarceration, especially when anticipating enforced separation soon after birth.

Goffman's (1959) mortification of self, although not specifically related to women, has echoes in the current study, illustrating how a pregnant prisoner is given wearable marks of shame, such as handcuffs, ill‐fitting clothes and lactation stains. This article argues for the concept of institutional ignominy, building on Goffman's premise of the process of mortification, in that the pregnant prisoner revisits the stages of mortification (e.g. entering the institution and being deprived of outside networks) many times as she transitions between prison and hospital during her pregnancy. Goffman's analysis of institutions described the structure and micro‐interactions as within a single, locatable trajectory, the mental asylum, noting aspects in common with other total institutions. The current study has identified that the pregnant woman in prison, regardless of the type of setting, is also caught between two institutions – both separate, yet both causing her to want to hide either her prisoner status or her pregnancy status due to institutional ignominy. The uniqueness of this finding suggests that pregnancy, and the resulting necessity of regular outings in public for health assessments, leads to supplementary suffering and shame for a woman different from any other type of prisoner experience.

## Data Availability Statement

The data that support the findings of this study are openly available in [https://uhra.herts.ac.uk] at https://uhra.herts.ac.uk/handle/2299/20283,, reference number [2018‐07‐13T13:32:40Z].

## References

[shil13052-bib-0001] Abbott, L.J. (2018) The Incarcerated Pregnancy: An Ethnographic Study of Perinatal Women in English Prisons. Hatfield: University of Hertfordshire.

[shil13052-bib-0002] Abbott, L.J . (2019) Escorting pregnant prisoners‐the experiences of women and staff. Prison Service Journal Issue, 241, 20.

[shil13052-bib-0003] Albertson, K. , O'Keefe, C. , Lessing‐Turner, G. , Burke, C. , *et al* (2012) Tackling Health Inequalities Through Developing Evidence‐Based Policy and Practice with Childbearing Women in Prison: A Consultation The Hallam Centre for Community Justice, Sheffield Hallam University and The Mother and Infant. York: Research Unit, University of York.

[shil13052-bib-0004] Balin, J. (1988) The sacred dimensions of pregnancy and birth, Qualitative Sociology, 11, 4, 275–301.

[shil13052-bib-0005] Barbour, R. (2013) Introducing Qualitative Research: A Student Guide to the Craft of Doing Qualitative. Thousand Oaks: Research: Sage Publications Ltd.

[shil13052-bib-0006] Bradfield, Z. , Duggan, R. , Hauck, Y. and Kelly, M. (2018) Midwives being ‘with woman’: an integrative review, Women and Birth, 31, 2, 143–52. 28807466 10.1016/j.wombi.2017.07.011

[shil13052-bib-0007] Brons, L.L . (2015). Othering, an Analysis. *Transcience*. Vol. 6, Issue 1.

[shil13052-bib-0008] Canales, M.K. (2000) Othering: toward an understanding of difference, Advances in Nursing Science, 22, 4, 16–31. 10.1097/00012272-200006000-00003 10852666

[shil13052-bib-0009] Chamberlen, A. (2015) Embodying prison pain: women's experiences of self‐injury in prison and the emotions of punishment, Theoretical Criminology., 20, 2, 205–19.

[shil13052-bib-0010] Chambers, A.N. (2009) Impact of forced separation policy on incarcerated postpartum mothers, Policy, Politics, & Nursing Practice, 10, 3, 204–11. 10.1177/1527154409351592 20022914

[shil13052-bib-0011] Corston, J . (2007) The Corston Report: A Report by Baroness Jean Corston of a Review of Women with Particular Vulnerabilities in the Criminal Justice System: The Need for a Distinct, Radically Different, Visibly‐led, Strategic, Proportionate, Holistic, Woman‐centred, Integrated approach. Home Office.

[shil13052-bib-0012] Council of Europe . Committee of Ministers. (2006). European Prison Rules. Council of Europe. Recommendation Rec. 2 of the Committee of Ministers to member states on the European Prison Rules.

[shil13052-bib-0013] Crawley, E. (2005) Institutional thoughtlessness in prisons and its impacts on the day‐to‐day prison lives of elderly men, Journal of Contemporary Criminal Justice, 21, 4, 350–63.

[shil13052-bib-0014] Crewe, B. (2009) The Prisoner Society: Power, Adaptation and Social Life in an English Prison. Oxford: Oxford University Press.

[shil13052-bib-0015] Crewe, B. , Liebling, A. and Hulley, S. (2015) Staff‐prisoner relationships, staff professionalism, and the use of authority in public‐ and private‐sector prisons, Law & Social Inquiry, 40, 2, 309–44.

[shil13052-bib-0016] Crewe, B. , Hulley, S. and Wright, S . (2017) The gendered pains of life imprisonment. British Journal of Criminology, 57, 6, 1359–78.

[shil13052-bib-0017] Easton, S. (2018) Older prisoners, gender, and family life, Ageing, Gender and Family Law, 19, 142–58.

[shil13052-bib-0018] Edge, D. (2006) Perinatal Health Care in Prison: A Scoping Review of Policy and Provision. Manchester: The Prison Health Research Network.

[shil13052-bib-0019] Ferszt, G.G. and Clarke, J.G. (2012) Health care of pregnant women in US state prisons, Journal of Health Care for the Poor and Underserved, 23, 2, 557–69. 22643607 10.1353/hpu.2012.0048

[shil13052-bib-0020] Fritz, S. and Whiteacre, K. (2016) Prison nurseries: experiences of incarcerated women during pregnancy, Journal of Offender Rehabilitation, 55, 1, 1–20.

[shil13052-bib-0021] Gardiner, A. , Daniel, B. , Burgess, C. and Nolan, L. (2016) The Rose Project: Best for Babies ‐ Determining and Supporting the Best Interests and Wellbeing of Babies of Imprisoned Mothers in Scotland. Stirling: University of Stirling.

[shil13052-bib-0022] Goffman, E . (1959). The Presentation of Self in Everyday Life. In: Garden City, NY: Doubleday Anchor.

[shil13052-bib-0023] Goffman, E. (1961) On the Characteristics of Total Institutions. (pp 1–124). Holt, Rinehart and Winston.

[shil13052-bib-0024] Goffman, E. (1968) Asylums: Essays on the Social Situation of Mental Patients and Other Inmates: AldineTransaction.

[shil13052-bib-0025] Gullberg, S. (2013) State of the Estate ‐ Women in Prison's Report on the Women's Custodial Estate 2011–12. London: Women in Prison.

[shil13052-bib-0026] Halliday, A. , Miller, C. and Peterson, J. (2017) “What are women's prisons for?” Gendered states of incarceration and history as an agent for social change, Museums & Social Issues, 1–11.

[shil13052-bib-0027] Hammersley, M. and Atkinson, P. (2007) Ethnography: Principles in Practice. Abingdon: Routledge.

[shil13052-bib-0028] Harvey, J . (2008). An Embedded Multimethod Approach to Prison Research. In King, R., and Wincup, E. (2008). Doing Research on Crime and Justice: Part V: Chapter 18: pp.487‐500. Oxford University Press.

[shil13052-bib-0029] Hochschild, A.R. (2012) The Managed Heart: Commercialization of Human Feeling. Berkeley: University of California Press. 3rd Edition.

[shil13052-bib-0030] Jewkes, Y. (2012) Autoethnography and emotion as intellectual resources doing prison research differently, Qualitative Inquiry, 18, 1, 63–75.

[shil13052-bib-0031] Kennedy, A. , Marshall, D. , Parkinson, D. , Delap, N. , *et al* (2016) Birth Charter for Women in Prisons in England and Wales. London: Birth Companions.

[shil13052-bib-0032] Laing, R . (1960) The Divided Self: An Existential Study in Sanity and Madness (Vol. 731) Penguin UK.

[shil13052-bib-0033] Leder, D. (2016) The Distressed Body: Rethinking Illness, Imprisonment, and Healing. Chicago: University of Chicago Press.

[shil13052-bib-0034] Liebling, A. (1999) Doing research in prison: breaking the silence?, Theoretical Criminology, 3, 2, 147–73.

[shil13052-bib-0036] Liebling, A. and Maruna, S. (2013) The Effects of Imprisonment. Abingdon: Routledge.

[shil13052-bib-0037] Lofland, J. and Lofland, L.H. (2006) Analyzing Social Settings. Belmont: Wadsworth Publishing Company.

[shil13052-bib-0038] Longhurst, R. (2001) Breaking corporeal boundaries: pregnant bodies in public places, Contested Bodies, 81–94.

[shil13052-bib-0039] McCourt, C. , Stevens, T. , Sandall, J. and Brodie, P. (2006) Working With Women: Developing Continuity of Care in Practice. The New Midwifery: Science and Sensitivity in Practice, pp.141‐65.

[shil13052-bib-0040] Ministry of Justice (2019) Population and Capacity Briefing for Friday 22nd March 2019. London: Ministry of Justice.

[shil13052-bib-0041] Molina, N. (2019) Motherhood, Spirituality and Culture. London: Routledge.

[shil13052-bib-0042] National Offender Management Services . (2014) Mother and Baby Units Prison Service Instruction (PSI) 49/2014 PI 63/2014. Available at https://www.justice.gov.uk/downloads/offenders/psipso/psi-2014/psi-49-2014-mother-and-baby-units.pdf (Last accessed 10 June 2019).

[shil13052-bib-0043] Nursing and Midwifery Order, 45 Statutory (2001) Department of Health. Available at http://www.legislation.gov.uk/uksi/2002/253/pdfs/uksi_20020253_en.pdf (Last accessed 10 May 2019).

[shil13052-bib-0044] Oakley, A. (2016) The sociology of childbirth: an autobiographical journey through four decades of research, Sociology of Health & Illness., 38, 5, 85–836. 10.1111/1467-9566.12400 26857343

[shil13052-bib-0045] O'Keefe, C. and Dixon, L . (2015). Enhancing Care for Childbearing Women and their Babies in Prison. Available at http://www.birthcompanions.org.uk/media/Public/Resources/Extpublications/FINAL_MBU_report_8th_December_2016.pdf (Last accessed 14 March 2018).

[shil13052-bib-0046] Price, S. (2005) Maternity services for women in prison: a descriptive study, British Journal of Midwifery, 13, 6, 362–8.

[shil13052-bib-0047] Ritchie, J. , Lewis, J. , Nicholls, C.M. and Ormston, R. (2013) Qualitative Research Practice: A Guide for Social Science Students and Researchers. Thousand Oaks: Sage.

[shil13052-bib-0048] Rogan, M. (2017) Human rights and correctional health policy: a view from Europe, International Journal of Prisoner Health, 13, 1, 3–9. 28299970 10.1108/IJPH-08-2016-0049

[shil13052-bib-0049] Ross, M.W. , Liebling, A. and Tait, S. (2011) The relationships of prison climate to health service in correctional environments: inmate health care measurement, satisfaction and access in prisons, The Howard Journal of Criminal Justice, 50, 3, 262–74.

[shil13052-bib-0050] Sandall, J. , Soltani, H. , Gates, S. , Shennan, A. , *et al* (2013) Midwife‐led continuity models versus other models of care for childbearing women, Cochrane Database Systematic Rev, 8, 8. 10.1002/14651858.CD004667.pub3 23963739

[shil13052-bib-0051] Schroeder, C. and Bell, J. (2005) Doula birth support for incarcerated pregnant women, Public Health Nursing, 22, 1, 53–8. 15670325 10.1111/j.0737-1209.2005.22108.x

[shil13052-bib-0052] Sikand, M. (2017) Lost Spaces: Is the Current Provision for Women Prisoners to Gain a Place in a Prison Mother and Baby Unit Fair and Accessible? The Griffins Society University of Cambridge Institute of Criminology.

[shil13052-bib-0053] Sykes, G.M. (1958/2007) The Society of Captives: A Study of a Maximum‐Security Prison. Princeton: Princeton University Press.

[shil13052-bib-0054] Van Gundy, A. and Baumann‐Grau, A. (2016) Women, Incarceration, and Human Rights Violations: Feminist Criminology and Corrections. Abingdon: Routledge.

[shil13052-bib-0055] Wahidin, A . (2004). Older Women in the Criminal Justice System: Running out of Time. Jessica Kingsley Publishers.

[shil13052-bib-0056] Walker, T. , Shaw, J. , Hamilton, L. , Turpin, C. , *et al* (2016) Supporting imprisoned women who self‐harm: exploring prison staff strategies, Journal of Criminal Psychology, 6, 4, 173–86.

[shil13052-bib-0057] Wismont, J.M. (2000) The lived pregnancy experience of women in prison, Journal of Midwifery and Women's Health, 45, 4, 292–300. 10.1016/s1526-9523(00)00034-9 10983427

[shil13052-bib-0058] Zamble, E. and Porporino, F.J. (2013) Coping, Behavior, and Adaptation in Prison Inmates. Berlin: Springer Science and Business Media.

[shil13052-bib-0059] Zimbardo, P. (2016) Revisiting the stanford prison experiment: a lesson in the power of situation, Perspectives on Contemporary Issues, 309.

